# Using Response Surface Analysis to Interpret the Impact of Parent–Offspring Personality Similarity on Adolescent Externalizing Problems

**DOI:** 10.1002/per.2088

**Published:** 2017-01-12

**Authors:** Aart Franken, Odillia M. Laceulle, Marcel A.G. Van Aken, Johan Ormel

**Affiliations:** ^1^ Department of Developmental PsychologyUtrecht UniversityUtrechtThe Netherlands; ^2^ Department of Medical and Clinical PsychologyTilburg UniversityTilburgThe Netherlands; ^3^ University Medical Center GroningenUniversity of GroningenGroningenThe Netherlands

**Keywords:** antisocial behavior, dyadic and group analysis, social and personal relationships, statistical methods

## Abstract

Personality similarity between parent and offspring has been suggested to play an important role in offspring's development of externalizing problems. Nonetheless, much remains unknown regarding the nature of this association. This study aimed to investigate the effects of parent–offspring similarity at different levels of personality traits, comparing expectations based on evolutionary and goodness‐of‐fit perspectives. Two waves of data from the TRAILS study (*N* = 1587, 53% girls) were used to study parent–offspring similarity at different levels of personality traits at age 16 predicting externalizing problems at age 19. Polynomial regression analyses and Response Surface Analyses were used to disentangle effects of different levels and combinations of parents and offspring personality similarity. Although several facets of the offspring's personality had an impact on offspring's externalizing problems, few similarity effects were found. Therefore, there is little support for assumptions based on either an evolutionary or a goodness‐of‐fit perspective. Instead, our findings point in the direction that offspring personality, and at similar levels also parent personality might impact the development of externalizing problems during late adolescence. © 2017 The Authors. *European Journal of Personality* published by John Wiley & Sons Ltd on behalf of European Association of Personality Psychology

Personality has been associated with the development of externalizing problems (e.g. Malmberg et al., 2012; Manders, Scholte, Janssens, & De Bruyn, 2006; Oldehinkel, Hartman, De Winter, Veenstra, & Ormel, 2004; Ormel et al., 2005; Van Aken & Dubas, 2004; Van Tuijl, Branje, Dubas, Vermulst, & van Aken, 2005). Recently, studies of the association between personality and externalizing problems have started to examine personality within the broader social context (see also Back & Vazire, 2015). That is, individuals might be affected by their own personality, but also by the personality of important others and by the match between both personalities. Whereas various studies have examined personality similarity between peers and romantic partners (e.g. Robins, Caspi, & Moffitt, 2000; Selfhout et al., 2010), little is known regarding personality similarity between parents and offspring. Only two previous studies examined parent–offspring personality similarity (Langenhof, Komdeur, & Oldehinkel, 2015; Van Tuijl et al., 2005), suggesting that personality similarity might play an important role in offspring's development. However, it is not known whether the level of personality traits affects the impact of personality similarity. It is possible that similarity has a different impact at low or high levels of personality traits, or might differ depending whether the parent or the offspring has a higher level of certain personality traits.

Personality has been characterized as ‘relatively stable individual differences in affect, behaviour, and cognition’ (Denissen, 2014, p. 213). The Big Five model (Caspi, Roberts, & Shiner, 2005; McCrae & Costa, 1987) captures such individual differences in five traits: Extraversion, Agreeableness, Conscientiousness, Neuroticism, and Openness to experience. Of these traits, Extraversion, Neuroticism, and Openness have most consistently been associated with negative outcomes (e.g. Kotov, Gamez, Schmidt, & Watson, 2010). Studies have indicated that Extraversion was not or only moderately positively associated with externalizing problems (such as aggression, antisocial behavior, or delinquency), Openness was not or negatively associated with externalizing problems, and Neuroticism was positively associated with externalizing problems (John, Caspi, Robins, Moffitt, & Stouthamer‐Loeber, 1994; Jones, Miller, & Lynam, 2011; Klimstra, Akse, Hale, Raaijmakers, & Meeus, 2010; Miller & Lynam, 2001; Miller, Lynam, & Leukefeld, 2003).

The two studies investigating parent–offspring similarity in personality indicated that, when similarity mattered, it was associated with fewer externalizing problems (Langenhof et al., 2015; Van Tuijl et al., 2005). From an evolutionary perspective, such similarity might be beneficial. As personality is heritable, genetic factors account for approximately 40%–60% of individual differences in personality (e.g. Spinath & O'Connor, 2003), similarity in personality might indicate genetic similarity. From an evolutionary perspective, fathers would have thus more proof that they are the genetic father of their offspring and might therefore be more inclined to help kin that have similar personality characteristics (see Alvergne, Faurie, & Raymond, 2009; Dubas & van Aken, 2004; Geary, 2000).

The two previous studies investigating similarity used Q‐correlations (Van Tuijl et al., 2005) and difference scores (Langenhof et al., 2015). Such analyses do not differentiate between pairs who have similarly low or high personality traits. For example, offspring and parents who are both low on Neuroticism or both high on Neuroticism would receive the same score—indicating a high similarity. Moreover, such studies did not differentiate between the offspring or parent scoring higher or lower on a certain trait. Thus, a parent with higher Neuroticism than the offspring would have received the same difference score compared to offspring having higher Neuroticism than the parent does. Little is known, however, about whether the level of personality traits affects the impact of personality similarity; thus, whether effects differ for pairs who score similarly low or high on personality traits. From a goodness‐of‐fit perspective, an individual's temperament should match the demands and expectations of the social environment (Lerner, 1984; Seifer, 2000; Thomas & Chess, 1977). Similarity in positive traits, such as Extraversion or Openness, might lead to a better mutual understanding. Such similarity might thus be associated with better outcomes for the offspring, such as less externalizing problems. However, from an interpersonal circumplex perspective (see, Dryer & Horowitz, 1997; Wiggins, 1996) dissimilarity, or complementarity, might be preferable for some negative personality characteristics. In line with a goodness‐of‐fit perspective (Lerner, 1984; Seifer, 2000; Thomas & Chess, 1977), similarity at high levels of negative traits might lead to a bad fit between parent and offspring as the demands and expectations between the offspring's temperament and social environment (i.e. the parent) are suboptimal. Therefore, similarity at high levels of negative traits, such as Neuroticism, might be associated with a worse fit and thus might be associated with more externalizing problems.

A promising way to overcome the methodological limitations of earlier studies investigating similarity in parent offspring personality is using polynomial regression analyses (see Nestler, Grimm, & Schönbrodt, 2015; Shanock, Baran, Gentry, Pattison, & Heggestad, 2010). Such analyses allow differentiating between effects based on similarity at lower or higher levels of certain personality traits. Moreover, such analyses allow differentiating between parents or offspring scoring higher on certain traits. However, polynomial regression analyses have not yet been used to examine the effects of parent–offspring similarity in personality.

This study aimed to investigate the effects of parent–offspring similarity at different levels of personality traits, comparing expectations based on evolutionary and goodness‐of‐fit perspectives. This study combined a confirmatory and exploratory approach. The hypotheses were theory driven and confirmatory, while the comparison of different models was exploratory as we did not have a priori expectations which specific models might best fit our data. It was expected that from an evolutionary perspective, (1a) similarity in personality characteristics was beneficial regardless of the type of personality trait or the level of the personality trait. However, from a circumplex or goodness‐of‐fit perspective, (1b) similarity in negative traits (i.e. Neuroticism) was expected to have a negative impact on externalizing problems. Last, from an evolutionary perspective, (2) similarity effects were expected to be stronger for fathers than for mothers.

## Methods

### Participants and procedure

This study is part of the TRacking Adolescents' Individual Lives Survey (TRAILS), an ongoing prospective cohort study based on a sample representative of the Dutch population, investigating the emotional, social, and mental development from preadolescence into adulthood. Parental informed consent was obtained after the procedures had been fully explained. Detailed information about sample selection and analysis of non‐response bias has been reported elsewhere (De Winter et al., 2005; Huisman et al., 2008). This study was based on data collected between September 2005 to December 2007 (Time 3, age 16, of the TRAILS study), and October 2008 to September 2010 (Time 4, age 19, of the TRAILS study). At Time 1, 2230 participants (mean age = 11.1, *SD* = 0.6) enrolled in the study of whom 2149 (96.4%; mean age 13.6, *SD* = 0.5) participated at Time 2, 1816 (81.4%; mean age 16.3, *SD* = 0.7) at Time 3, and 1881 (84.3%; mean age 19.1, *SD* = 0.6) at Time 4.

Participants included 1587 adolescents who had filled out the personality questionnaire at age 16.2 (*SD* = 0.7, 51.7% girls, Time 3 of the TRAILS study) along with both their biological mother and (self‐reported) biological father, from here on referred to as mother and father. This selection creates a relatively homogeneous group with respect to parental influence during childhood. At age 19.0 (*SD* = 0.5, 53.0% girls, Time 4 of the TRAILS study), 1488 participants (93.8%) filled out the externalizing problems questionnaire. Analyses were thus based on 1587 participants starting at age 16, when personality was first assessed.

### Measures

#### Personality

(Age 16). To assess adolescent and parent personality, we used six facets of the Revised Neuroticism‐Extroversion‐Openness Personality‐Inventory (NEO‐PI‐R), measured at age 16. The NEO‐PI‐R is a personality questionnaire consisting of 30 facet scales covering the Five‐Factor Model of personality (Costa & McCrae, 1992). Due to time constraints during data collection, facets were a priori selected based on their relevance to behavioral problems (e.g. Jones et al., 2011). From the broad domain of Neuroticism, we used the facets anger hostility (Cronbach's *α* = .71), impulsivity (*α* = .51), and vulnerability (*α* = .77). Anger hostility relates to the tendency to experience anger and frustration, impulsiveness to low inhibition control and a strong activating response to cravings and urges, and vulnerability to susceptibility to stress. From the broad domain of Extraversion, we used the facets assertiveness (*α* = .75) and excitement‐seeking (*α* = .58). Assertiveness reflects social dominance; excitement‐seeking the need for high‐intensity stimulation. From the broad domain of Conscientiousness, we used the facet self‐discipline (*α* = .76), which measures the capacity to begin and complete tasks despite distractions. Available answers ranged from 1 (fully disagree) to 5 (fully agree). After recoding reversed items, facet scores were each based on the mean of eight questions.

#### Externalizing problems

(Age 19). The Adult Self Report (ASR) has been widely used to assess self‐report symptom dimensions (Achenbach & Rescorla, 2001). Symptom dimensions in the externalizing domain (*α* = .89) that are covered by the ASR are aggression and delinquent behavior. The mean score of 29 items was used, based on a three‐point Likert scale as 0 (not true) to 2 (very or often true).

### Analysis strategy

In order to assess the joint impact of parent and offspring personality on externalizing problems, it is important to take levels of personality into account. Similarity patterns, also called fit patterns, have been developed to assess different types of similarity between two predictor variables. Such patterns are based on two main assumptions. First, there is an optimal match between two variables such as parent and offspring personality traits. Second, deviation from this optimal match leads to less optimal outcomes and bigger deviations will have more impact on the outcomes. Therefore, using similarity patterns, it can be estimated whether there is an optimal level of similarity in personality when predicting offspring's externalizing problems. Polynomial regression analysis can be used to compare several types of similarity patterns.

Polynomial regression analyses investigate linear effects of predictor variables, quadratic effects of predictor variables, and effects of the interaction between the predictor variables. Specifically, an intercept (*b*0), a linear (*b*1), and quadratic (*b*3) effect of the offspring, a linear (*b*2) and quadratic effect of the parent (*b*5), and an interaction between the linear effects of parent and offspring (*b*4) are estimated. Due to the combination of quadratic terms and an interaction term, interpretations of polynomial regressions are notoriously difficult. To facilitate interpretation, *Response Surface Analyses* have been developed (see Box & Draper, 1987; Edwards & Parry, 1993; Shanock et al., 2010).

**Figure 1:**
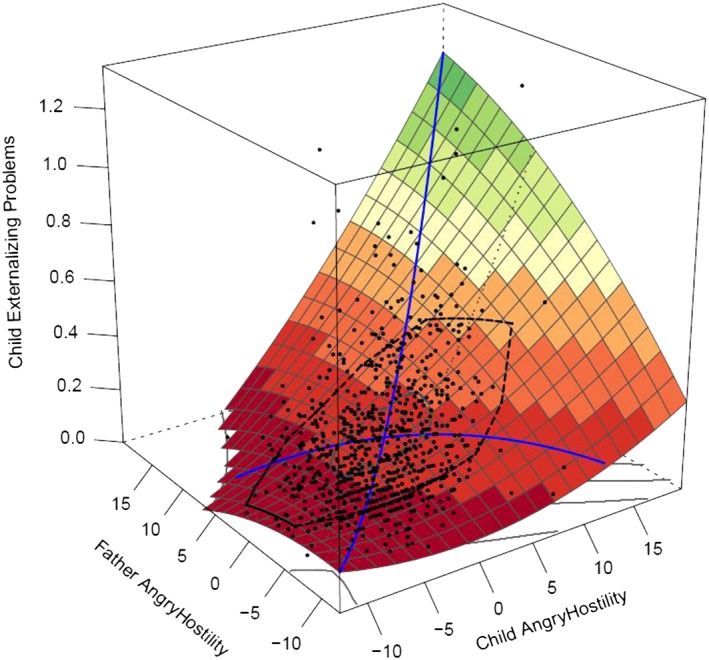
Full polynomial regression analysis: Father–offspring similarity in anger hostility is predicting externalizing problems. [Colour figure can be viewed at wileyonlinelibrary.com]

Response surface analyses provide a visual representation of the outcomes of polynomial regressions (see Figure 1), based on similarity (or congruence) and dissimilarity (or incongruence) between two variables. The x‐axis indicates the level of offspring's angry hostility, the y‐axis indicates the level of parent angry hostility, and the z‐axis indicates the level of offspring's externalizing problems. The dots in the figure represent participants, half of the participants are within the black line (bag plot) on the surface of Figure 1 and half of the participants are outside of this line. Two parameters (*a*1 and *a*2) assess effects among a Line of Congruence, or the line of similarity. The Line of Congruence is an imaginary line where parent and offspring have similar scores. For example, in Figure 1, this line would run from the near corner where both parent and offspring have low scores to the end at the far corner where both parent and offspring have high scores. These effects assess how externalizing behavior is associated with personality when parent and offspring have similar scores. They indicate a linear slope (*a*1) and quadratic slope (*a*2) of similarity of parent and offspring personality on externalizing problems. Thus, significant effects indicate that similarity of parent and offspring personality traits is associated with externalizing problems.

Other linear (*a*3) and quadratic (*a*4) terms indicate whether there is a dissimilarity effect of personality on externalizing problems, along a Line of Incongruence. This Line of Incongruence runs from the left corner where parents score high and offspring cores low, to the right corner where offspring scores high and parents score low. The linear slope effect (*a*3) indicates the likelihood for higher externalizing problems when the offspring scores higher than the parent on a personality trait. The quadratic effect (*a*4) indicates whether externalizing problems are especially likely at high or low levels of dissimilarity. Thus, significant effects indicate that dissimilarity in personality impacts externalizing problems.

One potential problem of polynomial regressions, however, is overfitting the data. Therefore, Schönbrodt (2016) suggested five simpler fit models, which are nested under the full polynomial model and use fewer degrees of freedom. Two of these fit models are mainly targeted at incommensurable measures (i.e. not measured on a similar scale) or used when there are theoretical expectations that the variables have a dissimilar impact on the outcome variable. As that is not the case for this study, these two models were disregarded. The other three fit models were compared with the full polynomial regression model and regular regression models.

The first types of models assume that there is no main effect of parent or offspring personality on the outcome variable, but allow for (dis)similarity effects. Thus, the level of the personality trait does not affect externalizing problems, but it does matter how (dis)similar parent and offspring are in personality characteristics. These models are thus in line with assumptions based on the evolutionary model that similarity in personality affects externalizing problems regardless of the level of personality traits. The sub model *shifted squared difference model* (*SSDQ*) models an effect of (dis)similarity, but optimal levels of (dis)similarity do not have to be at numerical equality. Thus, this model takes into account that the optimal match might not be when both parent and offspring have exactly the same score but allows the optimal match to be off from the numerical equality (for example if the optimal match is when offspring scores higher than parents).

The second types of fit models also assume (dis)similarity effects, but they also take the impact of the level of parent and offspring personality on externalizing problems into account. Thus, it also models how at similar levels of personality these traits are associated with externalizing problems. First, the sub model *basic rising ridge model (RR)* assumes that there is a main effect of (dis)similarity but also an effect of personality at similar levels of parents and offspring personality when predicting externalizing problems. Again, the *shifted version* of the rising ridge model (*SRR*) takes into account that the optimal match might not be when both parent and offspring have the exact same score.

These effects were estimated using the RSA package in R (Schönbrodt, 2015), guidelines from Schönbrodt (2016) were used for model selection. The main determinant for model selection was the corrected Akaike Information Criterion (AICc). Models with smaller AICc better fit the data. These weights can be compared using model weights called ‘Akaike weights’, which give the probability that a model is the best model of the candidate models, difference scores higher than two indicate significantly worse model fits. As AICc indices only indicate whether models are better compared to other models, rather than the absolute plausibility of models, *R* ^*2*^_*adj*_ should be used to assess the explained variance. If the explained variance (*R* ^*2*^_*adj*_) is significant, results can be interpreted. All variables were centered to facilitate interpretation. A score of zero thus means that participants had an average score, within their role (i.e. father, mother, or offspring). Positive scores indicate scoring higher than average on a personality trait while negative scores indicate scoring lower than average.

## Results

Table 1 shows the correlations between the main study variables, indicating that there was a significant, albeit modest, correlation between offspring's personality facets and the same father's or mother's personality facets; correlations ranged from 0.10 to 0.23. Offspring's, mother's, and father's anger hostility, impulsiveness, and excitement‐seeking were positively correlated to offspring's externalizing problems, and self‐discipline was negatively correlated to offspring's externalizing problems. Vulnerability was significantly positively correlated to offspring's externalizing problems for offspring and mother, but not for father, and assertiveness was not correlated to offspring's externalizing problems for offspring, mother, or father.

**Table 1:** Correlations between the main study variables

	*Mean* (*SD*)	1.	2.	3.	4.	5.	6.	7.	8.	9.	10.	11.	12.	13.	14.	15.	16.	17.	18.	19.
*Offspring variables*																				
1. Externalizing problems	0.22 (0.20)	1.00																		
2. Anger Hostility	19.88 (4.29)	0.39**	1.00																	
3. Assertiveness	23.90 (4.52)	0.05	−0.04	1.00																
4. Impulsiveness	23.20 (3.57)	0.34**	0.46**	−0.04	1.00															
5. Excitement‐Seeking	27.76 (4.16)	0.16**	0.03	0.32**	0.17**	1.00														
6. Self‐Discipline	26.20 (4.57)	−0.30**	−0.36**	0.23**	^−0.43**^	−0.05	1.00													
7. Vulnerability	19.29 (4.08)	0.23**	0.47**	−0.40**	0.40**	−0.18**	−0.47**	1.00												
*Mother variables*																				
8. Anger Hostility	19.00 (3.79)	0.13**	0.10**	−0.06	0.09**	0.00	−0.09**	0.07*	1.00											
9. Assertiveness	22.25 (4.81)	0.02	−0.08*	0.16**	0.00	0.04	0.06*	−0.12**	−0.05	1.00										
10. Impulsiveness	22.67 (3.74)	0.11**	0.11**	−0.01	0.15**	0.05	−0.06	0.07*	0.37**	0.03	1.00									
11. Excitement‐Seeking	20.81 (3.96)	0.06*	−0.02	0.08*	0.03	0.15**	−0.02	−0.03	0.05	0.21**	0.25**	1.00								
12. Self‐Discipline	29.09 (3.64)	−0.12**	−0.12**	0.09**	−0.10**	−0.01	0.13**	−0.11**	−0.35**	0.31**	−0.35**	−0.01	1.00							
13. Vulnerability	18.58 (3.70)	0.09**	0.12**	−0.11**	0.10**	−0.04	−0.11**	0.14**	0.46**	−0.47**	0.25**	−0.13**	−0.60**	1.00						
*Father variables*																				
14. Anger Hostility	19.24 (3.99)	0.13**	0.14**	−0.07	0.10**	0.01	−0.08*	0.14**	0.05	−0.06	0.09*	0.06	−0.07	0.06	1.00					
15. Assertiveness	24.75 (4.58)	0.02	−0.04	0.11**	−0.03	0.06	0.07*	−0.10**	−0.08*	0.06	0.00	0.02	0.00	−0.05	−0.13**	1.00				
16. Impulsiveness	22.52 (3.63)	0.17**	0.12**	−0.01	0.12**	0.03	−0.10**	0.09**	0.06	0.00	0.17**	0.13**	−0.06	0.04	0.37**	0.04	1.00			
17. Excitement‐Seeking	22.58 (4.20)	0.14**	0.01	0.10**	0.04	0.23**	0.00	0.00	0.01	0.05	0.17**	0.27**	−0.04	−0.03	0.03	0.27**	0.34**	1.00		
18. Self‐Discipline	29.47 (3.89)	−0.09*	−0.08*	0.07*	−0.02	0.00	0.11**	−0.08*	−0.09*	0.07	−0.05	−0.04	0.13**	−0.15**	−0.38**	0.43**	−0.31**	0.05	1.00	
19. Vulnerability	17.50 (3.62)	0.02	0.04	−0.07*	0.04	−0.04	−0.07	0.11**	0.06	−0.03	0.04	0.03	−0.07	0.07*	0.50**	−0.54**	0.23**	−0.17**	−0.64**	1.00

*Note:*

* *p* < .05.

** *p* < .01.

### Externalizing problems

Table 2 indicates that offspring externalizing problems was explained by all personality facets, as indicated by significant adjusted *R* ^*2*^_*adj*_ effects. Effect sizes range from 0.006 for the model including father's assertiveness to 0.185 for the model including father's anger hostility.

**Table 2:** Outcomes of the fit‐analyses of offspring and parent personality predicting externalizing problems

Personality facet	Best model	*R* ^2^_*adj*_
Anger hostility (see Table 3a for more details)		
Mother offspring	Full polynomial model	0.179***
Father offspring	Full polynomial model	0.185***
Impulsivity (see Table 3b for more details)		
Mother offspring	Only offspring effects	0.123***
Father offspring	Full polynomial model	0.156***
Vulnerability (see Table 3c for more details)		
Mother offspring	Only offspring effects	0.066***
Father offspring	Only offspring effects	0.066***
Assertiveness (see Table 3d for more details)		
Mother offspring	Rising Ridge model	0.007***
Father offspring	Only offspring effects	0.006**
Excitement seeking (see Table 3e for more details)		
Mother offspring	Only offspring effects	0.031***
Father offspring	Full polynomial model	0.043***
Self‐discipline (see Table 3f for more details)		
Mother offspring	Full polynomial model	0.107***
Father offspring	Only offspring effects	0.096***

*Note:*

** *p* < .01.

*** *p* < .001.

**Figure 2:**
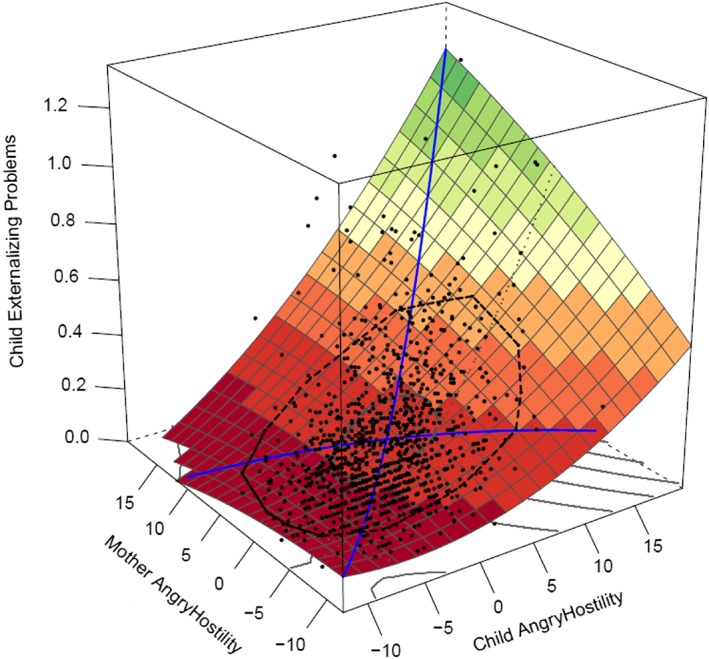
Full polynomial regression analysis: Mother–offspring similarity in anger hostility is predicting externalizing problems. [Colour figure can be viewed at wileyonlinelibrary.com]

Effects of *anger hostility.* Mother–offspring and father–offspring similarity on externalizing problems was best modeled (see Table 3a) by full polynomial regression models (mother effects: *a*1 = 0.023, *SE* = 0.002, *p* < .001; *a*2 = 0.001, *SE* = 0.001, *p* = .018; *a*3 = 0.013, *SE* = 0.002, *p* < .001; *a*4 = 0.000, *SE* = 0.001, *p* = .489 (*n.s.*); father effects: *a*1 = 0.022, *SE* = 0.002, *p* < .001; *a*2 = 0.001, *SE* = 0.001, *p* = .014; *a*3 = 0.013, *SE* = 0.001, *p* < .001; *a*4 = −0.001, *SE* = 0.001, *p* = .083 (*n.s.*)). Figure 1 shows these outcomes for the father–offspring effects; mother–offspring effects were similar (see Figure 2). The x‐axis indicates the level of offspring's angry hostility, the y‐axis indicates the level of parent angry hostility, and the z‐axis indicates the level of offspring's externalizing problems. The significant *a*1 and *a*2 effects indicate effects along the line of similarity; there is a linear and quadratic prediction from similarity in anger hostility on externalizing problems. An increase in anger hostility, when both parent and offspring have similar scores, of both parent and offspring is associated with an increase in externalizing problems, and this increase in externalizing problems tends to escalate at higher levels of anger hostility. Along the line of dissimilarity, the *a*4 effect was non‐significant; the degree of dissimilarity did not impact externalizing behavior. However, as indicated by a positive *a*3 effect, the direction of dissimilarity did impact externalizing behavior. Effects were stronger when the offspring has higher anger hostility than the parent has rather than vice versa.

**Table 3a:** Model comparison for the prediction of externalizing problems by mother, father, and offspring anger hostility. Ordered by delta AICc

Model names	*K*	AICc	Delta AICc	CFI	*p R* ^2^	*R* ^2^_adj_
*Mother and offspring*						
Full polynomial model	7	46 748.820	0.000	1.000	0.000	0.179
Moderated regression	5	46 755.330	6.507	0.966	0.000	0.173
Only offspring effects, squared	4	46 759.170	10.346	0.946	0.000	0.168
Additive effects	4	46 761.290	12.471	0.938	0.000	0.167
Shifted Rising Ridge	5	46 763.180	14.359	0.934	0.000	0.167
	Estimate (*SE*)	*p* Value				
b0—intercept	0.211 (0.008)	0.000				
b1—offspring linear	0.018 (0.001)	0.000				
b2—parent linear	0.005 (0.002)	0.001				
b3—offspring squared	0.001 (0.000)	0.042				
b4—interaction parent and offspring	0.001 (0.000)	0.046				
b5—parent squared	0.000 (0.000)	0.503				
*Father and offspring*						
Full polynomial model	7	40 125.080	0.000	1.000	0.000	0.185
Moderated regression	5	40 130.120	5.040	0.971	0.000	0.180
Only offspring effects, squared	4	40 132.980	7.906	0.956	0.000	0.169
Additive effects	4	40 140.100	15.024	0.927	0.000	0.165
Shifted Rising Ridge	5	40 142.070	16.999	0.923	0.000	0.165
	Estimate (*SE*)	*p* Value				
b0—intercept	0.216 (0.008)	0.000				
b1—offspring linear	0.017 (0.001)	0.000				
b2—parent linear	0.005 (0.002)	0.005				
b3—offspring squared	0.001 (0.000)	0.100				
b4—interaction parent and offspring	0.001 (0.000)	0.008				
b5—parent squared	0.000 (0.000)	0.069				

*Notes: K*, number of parameters; AICc, corrected Akaike Information Criterion; CFI, Comparative fit index; *R* ^2^, variance explained of the model; pmodel, *p* value for explained variance of the model; *R* ^2^.adj, adjusted *R* ^2^. Model abbreviations: X + Y, Model with two linear main effects; X + Y + XY, Moderated regression.

**Table 3b:** Model comparison for the prediction of externalizing problems by mother, father, and offspring impulsivity. Ordered by delta AICc

Model names	*K*	AICc	Delta AICc	CFI	*p R* ^2^	*R* ^2^_adj_
*Mother and offspring*						
Only offspring effects, squared	4	44 404.880	0.000	0.997	0.000	0.123
Full polynomial model	7	44 407.390	2.511	1.000	0.000	0.124
Additive effects	4	44 411.570	6.688	0.958	0.000	0.119
Shifted Rising Ridge	5	44 412.010	7.125	0.961	0.000	0.120
Only offspring effects	3	44 412.650	7.767	0.946	0.000	0.117
	Estimate (*SE*)	*p* Value				
b0—intercept	0.209 (0.006)	0.000				
b1—offspring linear	0.019 (0.002)	0.000				
b2—parent linear	—	—				
b3—offspring squared	0.001 (0.000)	0.004				
b4—interaction parent and offspring	—	—				
b5—parent squared	—	—				
*Father and offspring*						
Full polynomial model	7	37 889.460	0.000	1.000	0.000	0.156
Moderated regression	5	37 893.520	4.055	0.970	0.000	0.153
Additive effects	4	37 908.850	19.387	0.888	0.000	0.133
Shifted Rising Ridge	5	37 910.740	21.282	0.883	0.000	0.133
Only offspring effects, squared	4	37 913.320	23.859	0.865	0.000	0.123
	Estimate (*SE*)	*p* Value				
b0—intercept	0.201 (0.008)	0.000				
b1—offspring linear	0.019 (0.002)	0.000				
b2—parent linear	0.007 (0.002)	0.000				
b3—offspring squared	0.001 (0.000)	0.062				
b4—interaction parent and offspring	0.002 (0.001)	0.006				
b5—parent squared	0.001 (0.000)	0.076				

*Notes: K*, number of parameters; AICc, corrected Akaike Information Criterion; CFI, Comparative fit index; *R* ^2^, variance explained of the model; pmodel, *p* value for explained variance of the model; *R* ^2^.adj, adjusted *R* ^2^.

**Table 3c:** Model comparison for the prediction of externalizing problems by mother, father, and offspring vulnerability. Ordered by delta AICc

Model names	*K*	AICc	Delta AICc	CFI	*p R* ^2^	*R* ^2^_adj_
*Mother and offspring*						
Only offspring effects, squared	4	19 532.760	0.000	0.994	0.000	0.066
Full polynomial model	7	19 535.250	2.486	1.000	0.000	0.067
Shifted Rising Ridge	5	19 542.630	9.861	0.896	0.000	0.062
Additive effects	4	19 547.000	14.235	0.836	0.000	0.057
Moderated regression	5	19 548.020	15.260	0.836	0.000	0.058
	Estimate (*SE*)	*p* Value				
b0—intercept	0.206 (0.006)	0.000				
b1—offspring linear	0.043 (0.006)	0.000				
b2—parent linear	—	—				
b3—offspring squared	0.013 (0.004)	0.001				
b4—interaction parent and offspring	—	—				
b5—parent squared	—	—				
*Father and offspring*						
Only offspring effects, squared	4	16 971.240	0.000	1.000	0.000	0.066
Full polynomial model	7	16 975.430	4.189	1.000	0.000	0.067
Moderated regression	5	16 982.330	11.084	0.899	0.000	0.064
Only offspring effects	3	16 986.450	15.212	0.829	0.000	0.055
Additive effects	4	16 988.460	17.217	0.818	0.000	0.055
	Estimate (*SE*)	*p* Value				
b0—intercept	0.206 (0.006)	0.000				
b1—offspring linear	0.043 (0.006)	0.000				
b2—parent linear	—	—				
b3—offspring squared	0.013 (0.004)	0.001				
b4—interaction parent and offspring	—	—				
b5—parent squared	—	—				

*Notes: K*, number of parameters; AICc, corrected Akaike Information Criterion; CFI, Comparative fit index; *R* ^2^, variance explained of the model; pmodel, *p* value for explained variance of the model; *R* ^2^.adj, adjusted *R* ^2^.

**Table 3d:** Model comparison for the prediction of externalizing problems by mother, father, and offspring assertiveness. Ordered by delta AICc

Model names	*K*	AICc	Delta AICc	CFI	*p R* ^2^	*R* ^2^_adj_
*Mother and offspring*						
Rising Ridge model	4	48 834.440	0.000	0.719	0.001	0.007
Full polynomial model	7	48 834.470	0.031	1.000	0.001	0.010
Only offspring effects, squared	4	48 834.660	0.224	0.698	0.002	0.006
Shifted Rising Ridge	5	48 835.160	0.719	0.746	0.001	0.008
SQD	3	48 835.440	1.003	0.531	0.002	0.006
	Estimate (*SE*)	*p* Value				
b0—intercept	0.209 (0.007)	0.000				
b1—offspring linear	0.001 (0.001)	0.092				
b2—parent linear	0.001 (0.001)	0.092				
b3—offspring squared	0.000 (0.000)	0.023				
b4—interaction parent and offspring	−0.001 (0.000)	0.023				
b5—parent squared	0.000 (0.000)	0.023				
	*Father and offspring*					
Only offspring effects, squared	4	41 853.920	0.000	1.000	0.003	0.006
Full polynomial model	7	41 857.670	3.744	1.000	0.006	0.007
mean	3	41 857.770	3.843	0.375	0.023	0.003
Only offspring effects	3	41 857.780	3.856	0.374	0.040	0.002
null	2	41 859.250	5.327	0.000	NA	0.000
	Estimate (*SE*)	*p* Value				
b0—intercept	0.211 (0.007)	0.000				
b1—offspring linear	0.003 (0.001)	0.054				
b2—parent linear	—	—				
b3—offspring squared	0.000 (0.000)	0.026				
b4—interaction parent and offspring	—	—				
b5—parent squared	—	—				

*Notes: k*, number of parameters; AICc, corrected Akaike Information Criterion; CFI, Comparative fit index; *R* ^2^, variance explained of the model; pmodel, *p* value for explained variance of the model; *R* ^2^.adj, adjusted *R* ^2^.

**Table 3e:** Model comparison for the prediction of externalizing problems by mother, father, and offspring excitement seeking. Ordered by delta AICc

Model names	*K*	AICc	Delta AICc	CFI	*p R* ^2^	*R* ^2^_adj_
*Mother and offspring*						
Only offspring effects, squared	4	46 400.270	0.000	1.000	0.000	0.031
Full polynomial model	7	46 404.070	3.805	1.000	0.000	0.031
Only offspring effects	3	46 407.020	6.757	0.823	0.000	0.025
Moderated regression	5	46 407.070	6.802	0.873	0.000	0.027
Additive effects	4	46 407.860	7.598	0.827	0.000	0.025
	Estimate (*SE*)	*p* Value				
b0—intercept	0.209 (0.006)	0.000				
b1—offspring linear	0.009 (0.001)	0.000				
b2—parent linear	—	—				
b3—offspring squared	0.001 (0.000)	0.002				
b4—interaction parent and offspring	—	—				
b5—parent squared	—	—				
*Father and offspring*						
Full polynomial model	7	40 368.330	0.000	1.000	0.000	0.043
Rising Ridge model	4	40 369.890	1.552	0.907	0.000	0.041
Shifted Rising Ridge	5	40 371.690	3.356	0.890	0.000	0.040
Mean	3	40 371.860	3.524	0.846	0.000	0.037
Additive effects	4	40 373.640	5.308	0.830	0.000	0.035
	Estimate (*SE*)	*p* Value				
b0—intercept	0.210 (0.007)	0.000				
b1—offspring linear	0.007 (0.002)	0.000				
b2—parent linear	0.006 (0.002)	0.001				
b3—offspring squared	0.001 (0.000)	0.001				
b4—interaction parent and offspring	0.000 (0.000)	0.320				
b5—parent squared	0.000 (0.000)	0.653				

*Notes: k*, number of parameters; AICc, corrected Akaike Information Criterion; CFI, Comparative fit index; *R* ^2^, variance explained of the model; pmodel, *p* value for explained variance of the model; *R* ^2^.adj, adjusted *R* ^2^.

**Table 3f:** Model comparison for the prediction of externalizing problems by mother, father, and offspring self‐discipline. Ordered by delta AICc

Model names	*K*	AICc	Delta AICc	CFI	*p R* ^2^	*R* ^2^_adj_
*Mother and offspring*						
Full polynomial model	7	46 919.490	0.000	1.000	0.000	0.107
Moderated regression	5	46 923.250	3.756	0.960	0.000	0.102
Additive effects	4	46 926.080	6.586	0.933	0.000	0.098
Shifted Rising Ridge	5	46 927.230	7.732	0.932	0.000	0.098
Only offspring effects, squared	4	46 928.090	8.599	0.919	0.000	0.097
	Estimate (*SE*)	*p* Value				
b0—intercept	0.221 (0.007)	0.000				
b1—offspring linear	−0.013 (0.001)	0.000				
b2—parent linear	−0.005 (0.002)	0.001				
b3—offspring squared	0.000 (0.000)	0.156				
b4—interaction parent and offspring	0.001 (0.000)	0.020				
b5—parent squared	−0.001 (0.000)	0.004				
	*Father and offspring*					
Only offspring effects, squared	4	40 816.710	0.000	1.000	0.000	0.096
Additive effects	4	40 818.230	1.519	0.992	0.000	0.096
Only offspring effects	3	40 818.760	2.048	0.980	0.000	0.094
Shifted Rising Ridge	5	40 818.760	2.048	0.995	0.000	0.097
Moderated regression	5	40 819.700	2.995	0.988	0.000	0.096
	Estimate (*SE*)	*p* Value				
b0—intercept	0.213 (0.006)	0.000				
b1—offspring linear	−0.013 (0.001)	0.000				
b2—parent linear	—	—				
b3—offspring squared	0.000 (0.000)	0.088				
b4—interaction parent and offspring	—	—				
b5—parent squared	—	—				

*Notes: k*, number of parameters; AICc, corrected Akaike Information Criterion; CFI, Comparative fit index; *R* ^2^, variance explained of the model; pmodel, *p* value for explained variance of the model; *R* ^2^.adj, adjusted *R* ^2^.

Effects of *impulsivity* for the mother–offspring similarity hypothesis were best modeled by offspring effects only (see Table 3b). There was a significant linear (*b*1 = 0.019, *SE* = 0.002, *p* < .001) and quadratic effect (*b*3 = 0.001, *SE* = 0.000, *p* = .004) of offspring's impulsivity on offspring's externalizing problems. Thus, independent of mother's impulsivity, offspring's impulsivity was positively associated with externalizing problems and the association increased at higher levels of impulsivity. Father–offspring similarity was best modelled by a full polynomial regression model (*a*1 = 0.026, *SE* = 0.002, *p* < .001; *a*2 = 0.003, *SE* = 0.001, *p <* .001; *a*3 = 0.012, *SE* = 0.003, *p* < .001; *a*4 = 0.000, *SE* = 0.000, *p* = .621 (*n. s.*)). The significant *a*1 and *a*2 effects indicate effects along the line of similarity; there was a linear and quadratic prediction from similarity in impulsivity on externalizing problems. An increase in impulsivity at similar levels of impulsivity of both father and offspring was associated with an increase in externalizing problems. Moreover, this effect tends to escalate at higher levels of impulsivity. Along the line of dissimilarity, the *a*4 effect was non‐significant. Therefore, the degree of dissimilarity did not impact externalizing behavior. However, as indicated by a positive *a*3 effect, the direction of dissimilarity did matter. Effects were stronger when the offspring has higher impulsivity than the parent has rather than vice versa.

Effects of *vulnerability*. Mother–offspring and father–offspring similarity on externalizing problems were best modelled by effects of the offspring's vulnerability only (see Table 3c). Both for the mother–offspring and the father–offspring model, there was a linear effect (*b*1 = 0.043, *SE* = 0.006, *p* < .001) and a quadratic effect (*b*3 = 0.013, *SE* = 0.00, *p* = .001) for offspring's vulnerability predicting externalizing problems. In sum, independent on the vulnerability of mother or father, offspring's vulnerability is positively associated with externalizing problems, and this tends to escalate at higher levels of vulnerability.

**Figure 3:**
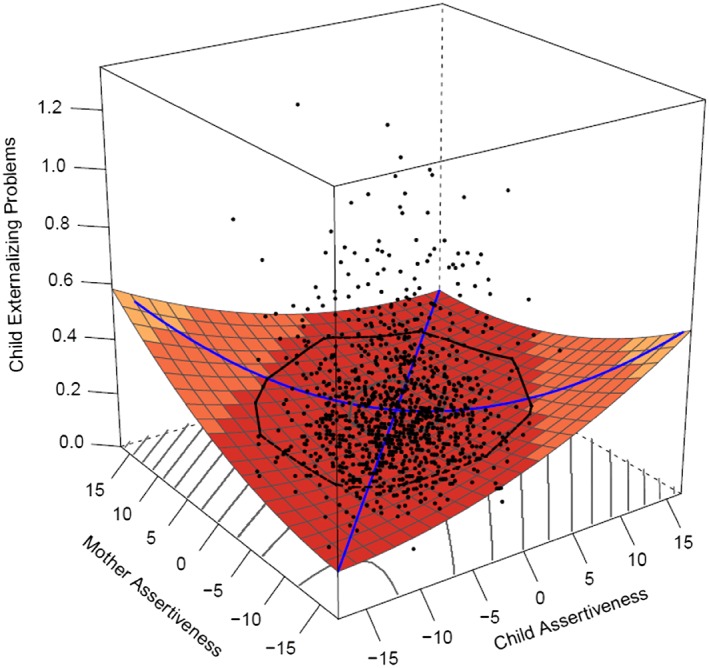
Rising Ridge model: Mother–offspring similarity in assertiveness is associated with less externalizing problems. [Colour figure can be viewed at wileyonlinelibrary.com]

**Figure 4:**
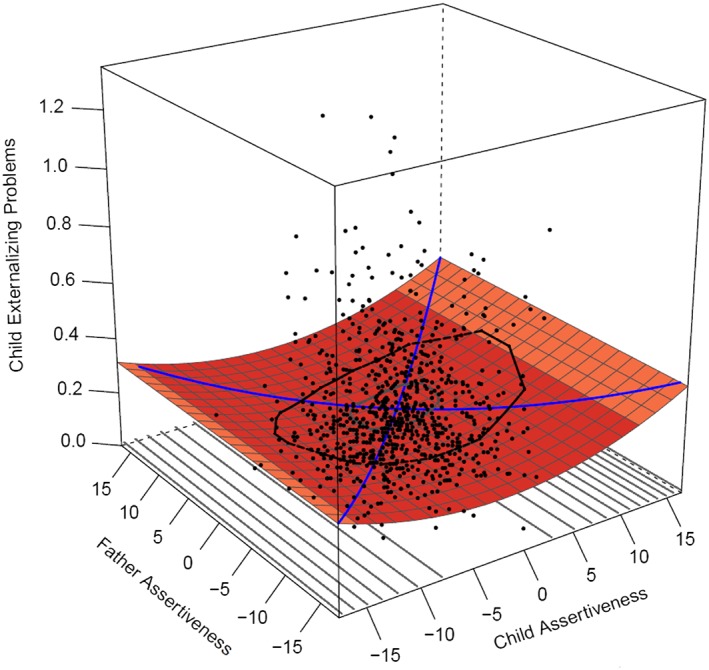
Full polynomial regression analysis: Father–offspring similarity in assertiveness predicting externalizing problems. [Colour figure can be viewed at wileyonlinelibrary.com]

Effects of *assertiveness* mother–offspring similarity was best modeled (see Table 3d) by a Rising Ridge model. Although the Rising Ridge model had the lowest AICc, other models such as the full polynomial model or offspring only effects were equally good candidate models; as the Delta AICc was less than two. This Rising Ridge model indicates that more similarity is associated with less externalizing problems (Figure 3), regardless of the level of assertiveness at which mother and offspring were similar. There was no significant linear (*a*1 = 0.003, *SE* = 0.002, *p* = .092 (*n.s.*)) or quadratic (*a*2 = 0.000, *SE* = 0.000, *p* = 1.000 (*n.s.*)) effect of assertiveness on externalizing problems along the line of similarity. Only the *a*4 effect was significant (*a*4 = 0.001, *SE* = 0.001, *p* = .023), indicating more similarity in assertiveness of mother and offspring is associated with less externalizing problems. Father–offspring effects of assertiveness were best modeled by offspring effects only. The linear effect was non‐significant (*b*1 = 0.03, *SE* = 0.054, *p* = .054 (*n.s.*)), but there was a small quadratic effect of offspring assertiveness on externalizing problems (*b*3 = 0.000, *SE* = 0.000, *p* = .026). This indicates that especially offspring with lower or higher levels of assertiveness experienced more externalizing problems compared to their peers who had average assertiveness (see Figure 4).

Effects of *excitement seeking*. Mother–offspring similarity on externalizing problems were best modeled (see Table 3e) by only offspring's effects. In this model, externalizing problems were significantly predicted by offspring's excitement seeking (*b*1 = 0.009, *SE* = 0.001, *p* < .001), and the quadratic effect of offspring's excitement seeking (*b*3 = 0.001, *SE* = 0.000, *p* = .002). Thus, independent on mother's excitement seeking, the offspring excitement seeking is positively associated with externalizing problems and has a tendency to escalate at higher levels of offspring excitement seeking. Effects of father–offspring similarity in excitement seeking on externalizing problems was best modeled by the full polynomial regression model (*a*1 = 0.013, *SE* = 0.002, *p* < .001; *a*2 = 0.000 *SE* = 0.000, *p* = .431 (*n.s.*); *a*3 = 0.002, *SE* = 0.003, *p* = .461 (*n.s.*); *a*4 = 0.001, *SE* = 0.001, *p* = .129 (*n.s.*)). The significant *a*1 effect indicates that along the line of similarity, there is a linear prediction from similarity in excitement seeking on externalizing problems. This effect was linear rather than quadratic, as indicated by the non‐significant *a*2 effect. Thus, at similar levels of excitement seeking, there was a positive association linear association between excitement seeking and externalizing problems. Along the line of dissimilarity, the *a*4 effect was non‐significant, thus the degree of dissimilarity did not impact externalizing behavior. Furthermore, as indicated by a non‐significant *a*3 effect, effects did not depend on the direction of dissimilarity. It did not matter whether the father or offspring had higher excitement seeking.

**Figure 5:**
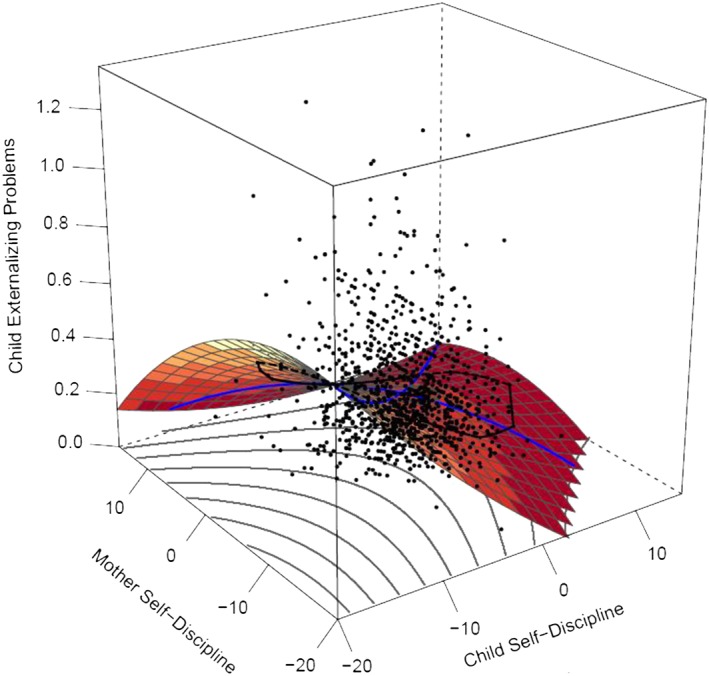
Full polynomial regression analysis: Mother–offspring similarity in self‐discipline predicting externalizing problems. [Colour figure can be viewed at wileyonlinelibrary.com]

Effects of *self‐discipline* mother–offspring similarity on externalizing problems were best modeled (see Table 3f) by a full polynomial regression model (*a*1 = −0.018, *SE* = 0.002, *p* < .001; *a*2 = 0.000, *SE* = 0.000, *p* = .304 (*n.s*)*.; a*3 = −0.008, *SE* = 0.002, *p* < .001; *a*4 = −0.001, *SE* = 0.00, *p* = .018). Figure 5 shows that the significant negative *a*1 and the non‐significant *a*2 effects indicate that there is a negative linear effect along the line of similarity of self‐discipline on externalizing problems. An increase in self‐discipline, while mother and offspring have similar self‐discipline, of mother and offspring is associated with a decrease in externalizing problems. The significant negative *a*3 effects indicate that the direction of difference in self‐discipline matters. Externalizing problems are more likely when the mother has higher self‐discipline compared to the offspring, rather than vice versa. The negative *a*4 effect, indicating the curvature along the line of dissimilarity, was also significant. This indicates that externalizing problems are especially likely when mother and offspring have a similar level of self‐discipline. For father–offspring similarity, only the offspring's self‐discipline predicted offspring's externalizing problems. There was a linear effect of offspring's self‐discipline on externalizing problems (*b*1 = −0.013, *SE* = 0.001, *p* < .001), but the quadratic effect was non‐significant (*b*3 = 0.000, *SE* = 0.000, *p* = .088 (*n. s.*)). Thus, independent of father's self‐discipline, offspring's self‐discipline is negatively associated with externalizing problems.

## Discussion

This study set out to explore the effects of parent–offspring personality similarity on externalizing problems. Hypotheses based on an evolutionary and on a goodness‐of‐fit perspective were compared, using sophisticated analyses and response surface plots. Findings indicated that, in contrast to earlier studies (e.g. Van Tuijl et al., 2005), similarity was mostly unrelated to offspring externalizing problems. One exception was offspring–*mother* similarity in assertiveness, a facet of Extraversion. Similarity between mother and offspring was associated with fewer externalizing problems, independent of the level of assertiveness. Notably, similarity in mother–offspring self‐discipline was negatively rather than positively associated with externalizing problems. With an increased similarity in self‐discipline for mother and offspring, the chance of later externalizing problems for the offspring increased. Hypotheses based on an evolutionary perspective therefore received no support: Similarity was not beneficial regardless of the trait or the level of the trait nor did similarity matter more for fathers than for mothers. There was limited support for the hypothesis based on a goodness‐of‐fit or interpersonal circumplex perspective. Although similarity was beneficial for a facet of Extraversion, a facet which is associated with lower externalizing problems, it was detrimental for a facet of Conscientiousness which is also associated with fewer externalizing problems. However, other findings indicated that both effects of parent's and offspring's personality matter, and at similar levels of personality these personality facets were associated with externalizing problems.

### Personality similarity and externalizing problems

Three facets of *Neuroticism* were investigated: anger hostility, impulsivity, and vulnerability. Offspring's Neuroticism predicted offspring's externalizing problems, in line with previous findings (e.g. Klimstra et al., 2010; Miller & Lynam, 2001). Based on the goodness‐of‐fit perspective, it was expected that similarity at higher levels of parent and offspring anger hostility was associated with more externalizing problems. However, rather than an effect of similarity, at similar levels of angry hostility of both parent and offspring predicted externalizing problems. Furthermore, externalizing problems were more likely when the offspring had higher anger hostility than the parent did rather than vice versa. Moreover, for mother–offspring impulsivity and both mother and father–offspring vulnerability, only the offspring's characteristics affected offspring's externalizing problems. Higher levels of impulsivity and vulnerability were associated with more externalizing problems. For father–offspring impulsivity, both father and offspring personality were associated with externalizing problems at similar levels of this facet. Some previous studies did not find a significant association between children's Neuroticism and externalizing problems (e.g. John et al., 1994). Possibly, especially parent's angry hostility is important in explaining the association between Neuroticism and offspring's externalizing problems. Broader indicators of Neuroticism might fail to detect effects based on more specific facets of personality. Angry hostility, impulsivity, and vulnerability have been associated with externalizing problems, while other facets of Neuroticism such as anxiety, or self‐consciousness have not always been associated with externalizing problems (e.g. Jones et al., 2011; Miller et al., 2003). In sum, for the facets anger hostility, impulsivity, and vulnerability of Neuroticism there were almost no differences between the findings for father–offspring and mother–offspring similarity, and higher scores of the offspring on all investigated Neuroticism facets were related to more externalizing problems.

Two facets of *Extraversion* were investigated: assertiveness and excitement seeking. In line with previous studies (e.g. John et al., 1994; Jones et al., 2011; Miller & Lynam, 2001), it was expected that Extraversion would be weakly or not associated with externalizing problems. In line with these expectations, assertiveness and excitement seeking only explained a small, although statistically significant, portion of externalizing problems. The explained variances were mainly based on offspring‐only effects, which means that the offspring's (and not the parent's) Extraversion predicted the offspring's future externalizing problems. Only for excitement seeking both father's and offspring's excitement seeking mattered, excitement seeking at similar levels for both father's and offspring's excitement seeking was positively associated with offspring's externalizing problems. One exception was mother–offspring similarity in assertiveness. Offspring who differed from their mother in their level of assertiveness were more likely to experience externalizing problems, compared to offspring who were more similar to their mother. There was, however, no direct effect of mother's or offspring's level of assertiveness in predicting externalizing problems. Therefore, the association between facets of Extraversion and offspring's externalizing problems depends both on the facet of Extraversion and the parent being studied.

One facet of *Conscientiousness* was studied: self‐discipline. Conscientiousness has been associated with less externalizing problems (e.g. John et al., 1994; Jones et al., 2011; Klimstra et al., 2010; Miller & Lynam, 2001; Miller et al., 2003). An increase in self‐discipline at similar levels of this facet of Conscientiousness for both mother and offspring was associated with less externalizing problems. Furthermore, externalizing problems were associated with similarity for conscientiousness. More rather than less similarity of self‐discipline was associated with externalizing problems. Moreover, the effects of offspring's self‐discipline were larger than the effects of mother's self‐discipline in explaining externalizing problems. Father's self‐discipline was not associated with offspring's externalizing problems. Thus, self‐discipline, a facet of Conscientiousness, was associated with less externalizing problems, and this was based on mother's and offspring's level of Conscientiousness.

In sum, rather than parent–offspring similarity, offspring's personality at the age of 16 seems to be most important in explaining offspring's externalizing problems at the age of 19. With an exception of the effects of mother and offspring in assertiveness, all facets of offspring's personality were associated with future externalizing problems. Similarity only predicted externalizing behavior for mother's and offspring's assertiveness and self‐discipline. For the other facets, both parents and offspring or only the offspring's personality was associated with offspring's externalizing problems. Therefore, there is little support for the two hypotheses based on an evolutionary perspective: Similarity in parent–offspring personality was mostly not beneficial, and for self‐discipline even detrimental, and mother–offspring rather than father–offspring similarity in personality was associated with externalizing problems. Possibly mothers' personality, rather than fathers' personality, has a higher impact on offspring's future externalizing problems as mothers, in general, spend more time with their offspring. In light of a goodness‐of‐fit or interpersonal circumplex perspective, only for a facet of Extraversion similarity was associated with less externalizing problems and for a facet of Conscientiousness similarity was even associated with more externalizing problems. Thus, only for one facet negatively associated with externalizing problems similarity was associated with less externalizing problems, while for another facet negatively associated with externalizing problems similarity was even detrimal. For other facets, similarity was not associated with externalizing problems. Thus, there was little support for the hypotheses based on a goodness‐of‐fit or interpersonal circumplex perspective. However, for quite some facets of personality traits, the offspring's personality mattered most in predicting externalizing problems. Therefore, regardless of parents' personality, the offspring personality affected future externalizing problems.

### Strengths and limitations

The current study was the first to use both polynomial regression analyses and similarity fit indices to identify how parent–offspring personality similarity best predicted offspring's externalizing problems. Polynomial regression analyses overcome two major shortcomings of earlier studies. First, similarity was not expected to be equal at different levels of personality traits; the impact of similarity at higher and lower levels of personality traits was allowed to be different. Second, different scores were not expected to be symmetrical, effects of parents having a higher score on a personality trait than their offspring was not expected to have the same impact as offspring having a higher score on a personality trait than the parent. Third, this study combined a confirmatory and exploratory approach. The hypotheses were theory driven and confirmatory, while the comparison of different models was exploratory as we did not a priori have expectations which specific models might best fit our data. Future studies might be able to also use confirmatory approaches to a priori identify the best way to model effects of parents offspring similarity. Furthermore, our findings were based on a large representative dataset of Dutch adolescents.

There were also some limitations to this study. First, as Van Tuijl et al. (2005) investigated early adolescents (around 13 years old), age differences between the samples might help explain why we did not find many effects of parent–offspring similarity. TRAILS data only assessed Big Five facets of parent and offspring personality when participants were 16 years old. Parents might have more influence on offspring during early rather than late adolescence. Second, this study did not control for externalizing behavior at age 16, while predicting externalizing behavior at age 19 as this is not possible in the current fSRM package. Moreover, the measures for externalizing problems differed between the assessments in the TRAILS study. As analyses were already highly complex, taking into account externalizing problems at age 16 may further complicate the model beyond the current aims of the study. Longitudinal studies investigating the interplay between parent and offspring personality and externalizing problems might shed light on more complex longitudinal developments. For example, parenting styles might have a different impact depending on offspring's personality (Prinzie et al., 2003). Moreover, offspring's problems might interact with the temperament of mothers (Atzaba‐Poria, Deater‐Deckard, & Bell, 2014). Also, testing multiple analyses in such a large sample increases the possibility of chance findings. However, we did take effect size into account to assess the magnitude of our findings. Last, underlying processes through which similarity in personality might impact externalizing behavior were not investigated. Future studies might aim to further disentangle this process, where it might be possible that dissimilarity in personality leads to a worse relationship quality, which in turn affects externalizing behavior development (see Van Tuijl et al., 2005). Although Van Tuijl et al. (2005) did not find relationship quality to impact effects based on personality similarity, possibly the use of complex analyses such as polynomial regression analyses might help uncover such processes.

### Conclusion

This study investigated different levels of parent–offspring personality similarity, and how these were associated with offspring's externalizing problems. From an evolutionary and a goodness‐of‐fit perspective similarity in personality was expected to be beneficial, at least at certain levels of traits. Therefore, they were expected to be associated with fewer externalizing problems for the offspring. However, hardly any similarity effects were found. Therefore, there is little support for assumptions based on either model. Although it might be argued that similarity leads to a more optimal match between parents and offspring, our findings point in the direction of additive effects of parent and offspring personality on the development of externalizing problems. Adolescents were especially likely to experience externalizing problems when both they and their parents had similar high levels of negative personality traits. Moreover, for several facets of personality, only the offspring's personality had an impact on offspring's externalizing problems. Therefore, interventions might identify adolescents based on their own personality as for almost all traits this had an impact on externalizing problems; including the personality of parents might only be beneficial when studying certain facets of personality traits.
